# Investigating machine learning algorithms to classify label-free images of pancreatic neuroendocrine neoplasms

**DOI:** 10.1117/1.BIOS.2.4.045001

**Published:** 2025-10-02

**Authors:** Noelle Daigle, Shuyuan Guan, Suzann Duan, Thomas G. Knapp, Eung-Joo Lee, Ali Azhdarinia, Sukhen C. Ghosh, Solmaz AghaAmiri, Servando Hernandez Vargas, Naruhiko Ikoma, Jeannelyn Estrella, Martin J. Schnermann, Tobias Else, Michelle Kang Kim, Juanita L. Merchant, Travis W. Sawyer

**Affiliations:** a University of Arizona, Wyant College of Optical Sciences, Tucson, Arizona, United States; b University of California, Irvine, Department of Pharmaceutical Sciences, Irvine, Arizona, United States; c University of Arizona, Department of Biomedical Engineering, Tucson, Arizona, United States; d University of Arizona, Department of Electrical and Computer Engineering, Tucson, Arizona, United States; e The University of Texas Health Science Center at Houston, Houston, Texas, United States; f The University of Texas MD Anderson Cancer Center, Houston, Texas, United States; g National Cancer Institute, Center for Cancer Research, Chemical Biology Laboratory, Frederick, Maryland, United States; h University of Michigan Medical School, Division of Metabolism, Endocrinology and Diabetes, Department of Internal Medicine, Ann Arbor, Michigan, United States; i Cleveland Clinic, Department of Gastroenterology, Hepatology, and Nutrition, Cleveland, Ohio, United States; j University of Arizona, College of Medicine, Tucson, Arizona, United States

**Keywords:** machine learning, artificial intelligence, pancreatic neuroendocrine neoplasms, multiphoton microscopy

## Abstract

**Significance:**

Pancreatic neuroendocrine neoplasms (PNENs) are an uncommon cancer whose incidence rate has increased dramatically in recent years. Surgery is the only potentially curative treatment, which relies on both preoperative tumor localization and postoperative margin definition using histopathological examination for decision making. If pathology could be automated, valuable time and resources could be saved.

**Aim:**

In this study, we investigate the ability of machine learning (ML) with handcrafted features, as well as deep learning, to classify label-free microscopy images of PNENs as a first step toward automated pathology of such tumors.

**Approach:**

Patient samples of two different preparation types were imaged, and ML and convolutional neural networks (CNNs) were developed to test the ability of such algorithms to classify PNENs.

**Results:**

Our classification algorithms were able to distinguish PNENs from normal tissue with high accuracy using multiphoton microscopy (MPM) images, regardless of sample preparation. Using a combined FFPE and fixed frozen dataset, we achieved an AUC value of 0.793 and an accuracy of 80.6% with ML, and an AUC value of 0.977 and an accuracy of 96.43% using CNNs.

**Conclusions:**

Label-free MPM combined with deep learning can provide fast, accurate classification of PNENs. With the ability to assess margins rapidly and potentially automatically, both disease recurrence and the need for resections after initial surgery could be reduced.

Statement of Discovery We demonstrated that label-free multiphoton microscopy and deep learning can be used to classify pancreatic neuroendocrine neoplasms with high accuracy. This is a step toward automated digital pathology for such tumors.

## 1 Introduction

Pancreatic neuroendocrine neoplasms (PNENs) are a part of a broader category of cancers known as gastroenteropancreatic neuroendocrine neoplasms (GEP-NENs). GEP-NENs arise from neuroendocrine cells, such as the islets of Langerhans in the pancreas, and are identified by their expression of neuroendocrine markers synaptophysin and chromogranin A.^1^^,^^2^ In general, PNENs are an uncommon cancer with a prevalence of 1 in 100,000 people. However, the incidence of PNENs has risen as much as eightfold in the last several decades.^3^^,^^4^ Examination of the American Surveillance, Epidemiology, and End Results (SEER) database has suggested that the incidence of small PNENs <2  cm has increased by over 700% in recent years due to improved diagnostic techniques,^5^ while PNENs exhibit the lowest five-year survival rate of any GEP-NEN at 27% to 37.6% between 1973 and 2007 across all stages.^6^^,^^7^

While varied interventions such as peptide receptor radionuclide therapy (PRRT), immunotherapy, and chemotherapy exist, surgery is the only potential curative treatment.^8^^,^^9^ Recent guidelines from The North American Neuroendocrine Tumor Society have suggested resection of tumors >2  cm, observing tumors <1  cm, and evaluating tumors 1 to 2 cm in size on an individual basis for surgical intervention.^10^ Lesions are localized using positron emission tomography (PET)/computed tomography (CT) prior to surgery if possible, although some tumors may be undetectable with this method, as available contrast agents (e.g., gallium-68 DOTATATE) are not perfectly sensitive to all PNENs. Smaller PNENs may also be below the resolution limit of PET/CT imaging.^11^^,^^12^ Thus, imaging modalities fail to effectively localize tumors in up to 40% to 60% of patients, in which case endoscopic ultrasound (EUS) with fine needle aspiration (FNA) or with fine needle biopsy (FNB) may be performed. EUS, especially when combined with other preoperative imaging modalities, can detect and diagnose up to 97% of PNENs.^13^^–^^15^

After EUS-FNA or -FNB is performed, the extracted tissue must undergo histopathological evaluation. Tissue specimens are typically fixed and then embedded, sectioned, and placed on slides before any further immunohistochemistry staining and imaging is performed. This is a time-consuming process that uses hazardous chemicals and requires highly trained pathologists.^16^ If imaging could be performed at the surgical bedside during or immediately after diagnostic EUS, valuable time and resources could be saved and patient treatment expedited. Importantly, evaluation of surgical margins following tumor resection becomes a critical consideration as positive margins increase the chance for disease recurrence. These margins are typically assessed using frozen section histopathological examination either during or post-surgery.^17^^,^^18^ With the ability to assess margins rapidly and potentially automatically, both disease recurrence and the need for resections after initial surgery could be reduced.

To achieve this goal, a rapid label-free imaging modality capable of microscopic resolution is required. Multiphoton microscopy (MPM) is such an imaging technique that offers many benefits over traditional fluorescence microscopy.^19^^,^^20^ It relies on the principle of two-photon excitation to target intrinsic fluorescent markers within tissue. In this regime, two photons must be incident on the sample simultaneously to induce fluorescence. As a consequence, only tissue within the focal volume is excited, hence there is increased resolution and decreased photodamage associated with this imaging modality compared to single photon analogs.^21^^,^^22^

**Fig. 1:**
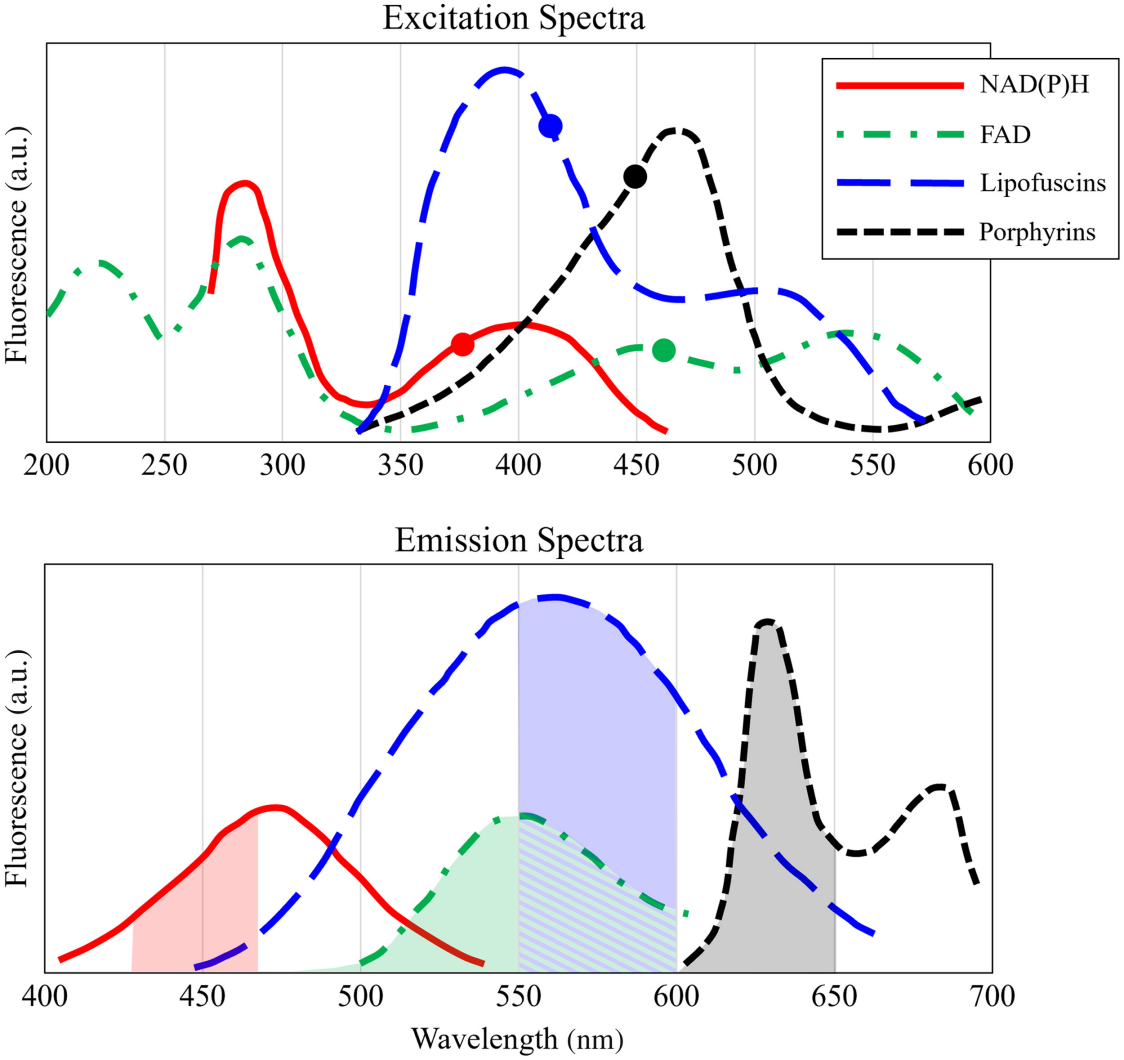
Excitation and emission spectra for each of the four fluorophores of interest: NADH(P)H, FAD, lipofuscins, and porphyrins. Derived from Monici et al.^25^ Specific excitation wavelengths used during this study are labeled on the excitation spectra plot using a circular marker. Values are for single photon excitation; actual excitation wavelengths used during imaging were double these values. Actual emission bands collected during imaging for each channel are colored in with their corresponding color on the emission spectra plot.

Endogenous fluorophores of particular interest for targeting with MPM in the scope of cancer are nicotinamide adenine dinucleotide (NAD(P)H), flavin adenine dinucleotide (FAD), porphyrins, and lipofuscins. NAD(P)H and FAD are coenzymes involved in cellular redox metabolism and are enriched in mitochondria and the cell cytoplasm.^23^ Porphyrins are present in the synthesis of blood constituents such as hemoglobin.^24^ Lipofuscins are pigments found during lipid oxidation,^25^ and are a marker of cellular senescence.^23^ Primary excitation and emission ranges for each of these four fluorophores are found in Fig. 1.^25^ As both neoplastic transformation and tumor growth involve changes to cellular metabolism, vascularization, and senescence,^26^ the fluorophores produced in these events may be used as biomarkers to distinguish tumor tissue from adjacent normal tissue.^23^^,^^24^

MPM is also capable of eliciting and collecting the second harmonic generation (SHG) signal. SHG is a second-order nonlinear optical response in a noncentrosymmetric material. In this process, two photons of the same wavelength incident simultaneously on the sample are converted into a photon of half the wavelength.^27^ Noncentrosymmetric materials exist within biological tissue and can produce observable SHG with no contrast agent or other modification. Of note are connective tissues that are primarily comprised of myosin, microtubules, and most importantly collagen,^28^ which produce a strong SHG signal and are frequently imaged as a method of label-free microscopy. While endogenous fluorescence can be elicited by analogous one-photon analogs, such as confocal microscopy, MPM is unique in simultaneously offering the ability to measure SHG and fluorescence.

Combining label-free microscopy and machine learning (ML) to enhance digital pathology is a relatively new yet promising field of research, and there are many groups endeavoring to improve both algorithm development and grow MPM datasets toward this goal.^29^^–^^31^ A previous study from our group demonstrated the feasibility of MPM to distinguish between duodenal neuroendocrine tumors (DNETs) and normal duodenal tissue in a digital pathology paradigm.^32^ DNETs are related to PNENs, both being a type of neuroendocrine tumor, and are similarly heterogeneous in presentation.^6^ While a promising proof of concept, significant differences between the organs require further testing of our approach on PNENs specifically.

Furthermore, this previous study relied on entirely formalin-fixed paraffin-embedded (FFPE) tissues, and the effect of tissue preparation was not assessed in this prior work, which is important to investigate, as surgical margin definition is typically performed with frozen specimens.^11^^,^^18^ Tissue samples stored in biobanks or pathology archives are generally prepared in one of two ways: fixed frozen or FFPE.^33^ Although protocols may differ, tissues are typically first fixed in neutral buffered formalin in both cases.^34^^–^^36^ Tissues then usually undergo dehydration in either sucrose under fixed frozen protocols,^35^ or ethanol in the case of FFPE protocols.^34^ Finally, tissues are embedded in either cryopreservation resin or paraffin wax, respectively, to then be sectioned and mounted.^34^^–^^36^ All steps of the preparation process may have an effect on the fluorescence properties of the tissues, depending on factors such as timing, temperature, and composition of chemicals used;^19^^,^^37^ autofluorescence in general has been noted to be increased by the fixation process and to increase with storage duration for FFPE samples.^38^^,^^39^ There has also been significant disagreement regarding the alteration of the fluorescence spectra of endogenous fluorophores such as NADH and FAD during fixation.^40^^–^^42^ These factors necessitate the study of the effect of sample preparation on the performance of a digital pathology-based classification algorithm.

In this study, we investigate the use of label-free MPM in combination with deep learning-based decision making for automated diagnosis of histopathological sections. First, we conduct imaging of PNENs and normal pancreatic tissue using MPM at various wavelengths to probe autofluorescence properties of the two tissues. We then test the ability of both ML with handcrafted image features, and deep learning analysis techniques, to differentiate between the two tissue types and compare results from the two methods. Finally, we further examine the effect of sample preparation on our results to determine whether algorithms can be trained to be robust to different tissue preparations.

## 2 Methods

This work was conducted in two major parts. First, we tested the ability of an ML algorithm with handcrafted image features to classify MPM images of FFPE, and of combined FFPE and frozen, samples, before then developing and testing deep learning models for the same purpose. The main goals of the first part of the study were to confirm the basic ability of classification algorithms to distinguish between PNENs and normal tissue, and to determine whether sample preparation was a factor in the results. As we had a small set of fixed frozen samples, we chose to evaluate the performance of the fixed frozen samples combined with the FFPE samples in comparison to just the FFPE samples to reduce the possibility of overfitting if we trained classifiers on only fixed frozen samples. By comparing the results of the FFPE only and the combined FFPE and fixed frozen datasets, we could examine whether the addition of a new sample preparation to the FFPE dataset affected results, and if so, whether one preparation was preferred over the other by the algorithm. Then having done this preliminary analysis, the main goal of the second part of the study was to improve our ability to classify these samples using deep learning techniques.

### 2.1 Datasets

Samples were acquired from three independent institutional biorepositories: 12 FFPE tumor samples from the University of Michigan Endocrine Oncology Repository (IRB #HUM00115310) through a Materials Transfer Agreement with Dr. Tobias Else, 17 FFPE tumor samples from the Biorepository and Pathology Core at Icahn School of Medicine at Mount Sinai (IRB STUDY-12-00145), and the remaining 19 FFPE and all 14 fixed frozen samples from the University of Arizona Tissue Acquisition and Cellular/Molecular Analysis Shared Resource (IRB #0600000609). It is important to recognize that these samples were collected from patients over several years and processed in different facilities, and as such there is potentially variability introduced during sample processing for which we cannot account. However, both tumor and normal specimens were collected roughly equally from each biorepository, which should mitigate these effects. All specimens were cut in 5 to 7 micron thick sections and mounted on glass slides, and all samples were imaged with a coverslip overlaid. FFPE samples were dry mounted, and fixed frozen samples were wet mounted with Fluoromount-G^®^. Each specimen was verified to contain only tumor or only normal tissue by a pathologist at the institution where the tissue was originally collected. Table 1 shows the composition of the two datasets. The ratio of tumor to normal tissue slides was roughly 1:1 for both datasets, but the ratio of FFPE samples to fixed frozen samples was around 3.5:1.

**Table 1:** The two datasets used in this study and their respective counts of tumor and normal samples.

	Tumor	Normal	Total
FFPE Samples	27	21	48
Fixed Frozen Samples	7	7	14
Total	34	28	62

### 2.2 Multiphoton Imaging

All samples were imaged using a LSM880 NLO upright multiphoton microscope with a 20x objective in the Optical Imaging Core at the University of Arizona. A tunable laser and detector allowed for custom excitation wavelengths and emission bands. Pixel dwell time was 4.1 microseconds for all images. The average laser power was wavelength dependent and therefore differed for each channel: 2.45 W for the NADH channel, 1.80 W for FAD, 2.89 W for lipofuscin, 2.09 W for porphyrin, and 2.29 W for SHG. Laser repetition rate was 80 MHz. Image gain was adjusted manually for each channel and each sample to standardize image histogram across samples without causing significant pixel saturation.

In addition to SHG, the four fluorescent channels were targeted to elicit signal from the fluorophores mentioned above: FAD, NADH, lipofuscins, and porphyrins. Table 2 lists the excitation and emission wavelengths for each of these channels, which are compared to the ideal excitation wavelengths and emission bands in Fig. 1. Imaging order of the channels was generally consistent across samples, so any potential consequences of imaging, such as photobleaching, would be similar across all samples. Wavelengths were chosen to limit crosstalk between the channels, but some overlap is unavoidable due to broad emission of endogenous fluorophores. For this reason, while each channel is labeled by the fluorophore that is expected to have strong emission for that excitation wavelength, each channel has contribution from multiple sources.

**Table 2:** Excitation and emission wavelengths for each of the five MPM channels. Excitation wavelengths are for two-photon excitation.

Channel	Expected endogenous fluorophores	Excitation wavelength [nm]	Emission range [nm]
1	Collagen, NAD(P)H	750	425 to 465
2	FAD	920	475 to 600
3	Lipofuscins	830	550 to 600
4	Porphyrins	900	600 to 650
5	SHG	880	430 to 450

**Fig. 2:**
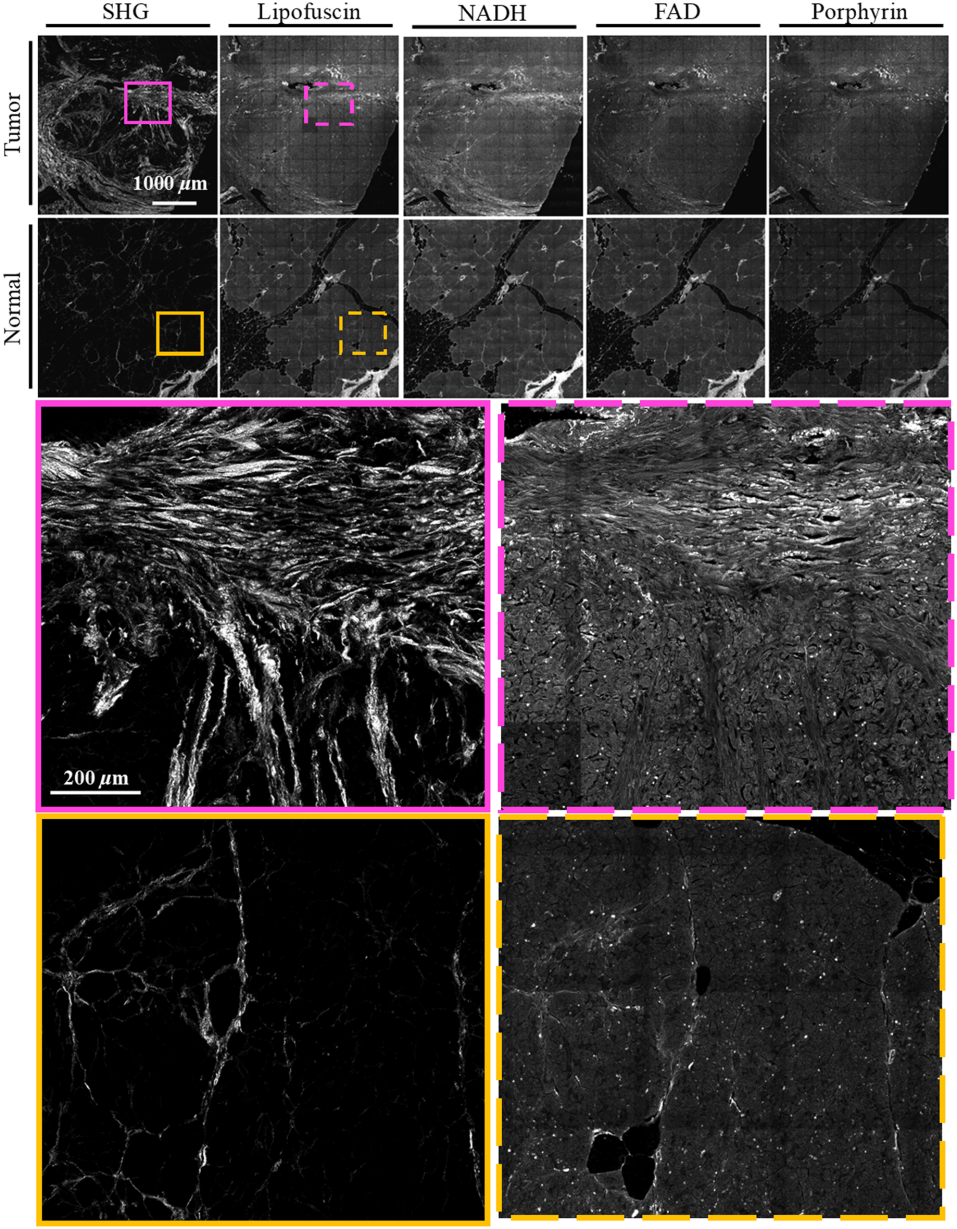
A representative tumor and normal sample for the FFPE preparation. The first and second rows show entire MPM images of the tumor and normal samples respectively. The third row shows a region of interest for the tumor sample on the SHG and lipofuscin images, and the fourth row shows the same for the normal sample. Images were uniformly brightened for ease of viewing.

**Fig. 3:**
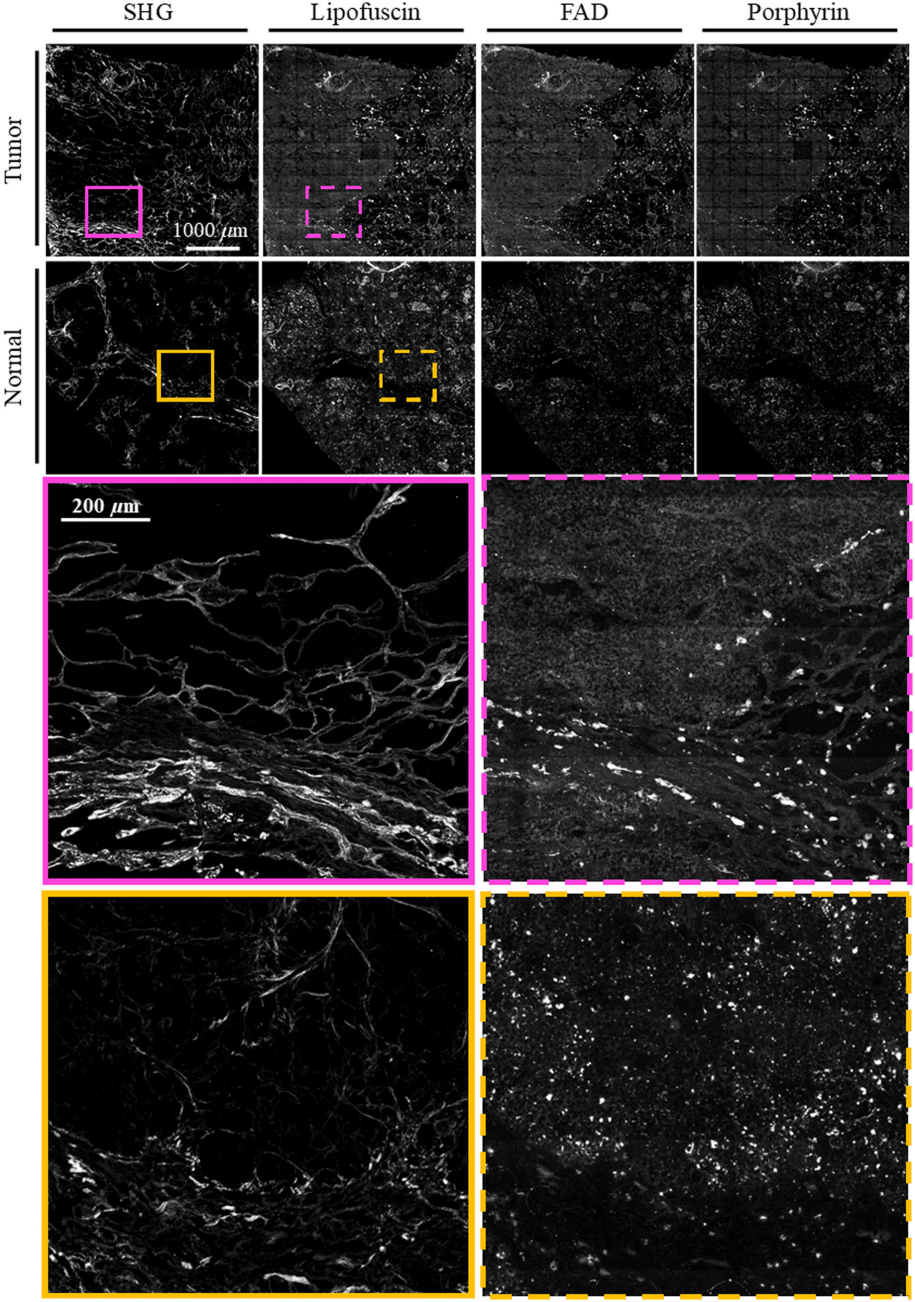
A representative tumor and normal sample for the fixed frozen preparation. The first and second rows show entire MPM images of the tumor and normal samples respectively. The third row shows a region of interest for the tumor sample on the SHG and lipofuscin images, and the fourth row shows the same for the normal sample. Note there is no NADH image for the fixed frozen samples due to interaction with the mounting medium at this wavelength. Images were uniformly brightened for ease of viewing.

For each sample, a region of interest was selected under the multiphoton microscope to limit scan duration to under 2 h. Imaging time was between 20 to 25 min for each channel. An automatic motorized stage collected images as grid of 13×13 tiles with 10% overlap. Individual tiles were 256×256  pixels with 16-bit depth. Images were taken in five z-stacks at 2 micron intervals to account for unevenly sectioned tissue or any tilt present in the sample. The whole image was assembled from tiles into stitched mosaics using techniques outlined in detail in Knapp et al.^43^ In short, tiles were first individually flat-field corrected. Tiles from each z-stack were then stitched using ImageJ’s Grid/Collection stitching plugin,^44^ and the max projection of these five z-stacks then taken to make one composite image. These whole reconstructed images were 3016×3016  pixels, covering an area of slightly less than 4  mm×4  mm on the sample, with the exception of those samples smaller than 4 mm in one or both dimensions. Figures 2 and 3 show representative MPM images of a tumor and normal sample of FFPE and fixed frozen sample preparations, respectively. The most apparent difference is in the SHG channel, where collagen signal is denser and brighter in both tumor samples compared to their normal counterparts.

While analyzing this data from the fixed frozen samples, it was noted that the Fluoromount-G^®^ mounting medium produces a small but detectable amount of fluorescence at the NADH excitation wavelength. The NADH channel was therefore omitted for any analysis involving fixed frozen samples. This interaction is documented in Supplementary Fig. 1.

### 2.3 Machine Learning Analysis

We first developed a classification algorithm using handcrafted image features along with linear discriminant analysis (LDA), which is well documented to perform well with a relatively low number of samples and a relatively high number of features.^45^ LDA is a supervised ML method which uses a linear combination of image features to classify data while also reducing dimensionality. In general LDA assumes the classes are normally distributed with a common covariance matrix. More specifically, we employ Fisher’s LDA which is optimized to maximize separation between classes and minimize separation between classes.^45^^,^^46^ The number of features fed into the LDA algorithm is an important consideration; the more features included, the higher the risk of overfitting and the longer the computation time. To mitigate these complications, we performed feature reduction before attempting LDA.

#### 2.3.1 Feature extraction

In order to perform ML analysis, we first extracted features from our images. As an initial step, we compared relative image brightness to determine if significant brightness differences between the tumor and normal sample populations existed and could be utilized as a feature. Then, Haralick texture features were calculated using the Mahotas library in Python.^47^ Published in 1973, Haralick texture features were originally designed to help quantify aerial images of crops; since then, these features have successfully been applied for analysis in biology and medicine as well.^48^^–^^50^ These features are based on the relationship between texture and tone. Haralick’s features use elements of gray level co-occurrence matrices (GLCMs) to calculate reference values for comparison to other images. GLCMs describe the relationship between adjacent pixels on an image specified by the distance between them, the angle between them, and pixels’ tone values. Our analysis used a pixel distance of 1 and averaged the four GLCM in each angular direction as the pancreas is fairly homogeneous and samples can be treated as rotationally symmetric. Entries in this averaged GLCM are then used to calculate Haralick’s texture features, listed in Supplementary Table 1. There are fourteen equations, although the fourteenth feature is often omitted because of its potential instability,^47^ and so we omitted this feature in our analysis. These features are not necessarily indicative of individual visible features in the image, but instead quantify overall spatial features in the image such as homogeneity and contrast. Features were calculated for each sample’s set of MPM images, which results in 65 features (13 features, 5 channels) for FFPE samples in the first part of the study, and 52 features (13 features, 4 channels) for combined FFPE and fixed frozen dataset in the second part. In this paper, features are referred to according to the MPM channel and which Haralick equation they correspond to; for example, SHG 01 is the first Haralick texture feature calculated on the SHG image for a sample.

#### 2.3.2 Feature reduction

First, the correlation between all features was calculated. To reduce the number of highly correlated features input to the LDA algorithm, we only retained features with a mean absolute correlation across all other features of less than 0.45 as this reduced the size of the feature set significantly and generally represents a low level of correlation in medical research.^51^^,^^52^ For the first part of the study, which used only FFPE samples, we reduced the number of features from 65 to 23 using this method, a 64.6% reduction. For the second part of the study, which used both FFPE and fixed frozen samples and did not use the NADH channel, we reduced the number of features from 52 to 23, a 55.8% reduction. Thresholds between 0.3 and 0.5 were also tested, but removed too few or too many features; for the FFPE only dataset, thresholds of 0.4 and 0.5 reduced the set to 13 and 36 features, respectively, for example. In addition to addressing overfitting concerns and computation time associated with LDA, reducing the number of features should also reduce crosstalk between imaging channels that were previously acknowledged.

After reduction, we reported the statistical significance of the remaining features. We first performed the Shapiro-Wilk test on each feature to determine which features were normally distributed.^53^ For those which were, we performed the Welch’s t-test, and for those which were not normally distributed, we performed a Mann-Whitney U test to determine if the tumor and normal samples had significantly different distributions.^54^^,^^55^ We then used the Benjamini-Hochberg procedure to adjust p-values and control the false discovery rate which can be significant when multiple comparisons are made.^56^ We chose to evaluate levels of significance at p<0.1, p<0.05, and p<0.02. These are slightly relaxed criteria to enable higher discovery rates of potentially useful features. This analysis was intended to understand whether statistical significance correlated with features chosen by the ML algorithm.

#### 2.3.3 Linear discriminant analysis

The LDA algorithm uses subsets of n=1,2,3,… features to attempt to classify the data, up to the maximum selected number of features. Subsets including more features can be expected to cause algorithm performance to plateau and eventually degrade due to overfitting.^32^ To determine the highest number of features we should give to the algorithm, we ran the algorithm testing each subset n until the algorithm performance began to plateau. For the FFPE dataset, this was n=5 features. For the combined FFPE and fixed frozen dataset, this was n=6 features. Then for each subset of n features up to the max, we ran the algorithm 100 times to ensure that the slight randomness inherent to the LDA algorithm did not affect our results.

We used leave-one-out cross-validation^46^ in order to determine the best features for classifying our data, testing each possible combination of subsets of features n. In each run, we had N samples in our dataset and therefore conducted N rounds of LDA. Each round, a different sample was left out of the set; the remaining N−1 samples were used as the training set to define LDA classifiers. The model then attempted to classify the sample which was left out. The final score of the algorithm for this feature subset is the average over all N rounds of its ability to classify the sample left out correctly. Whichever subset of features performed the best for that particular n is the selected feature subset; this was repeated 100 times for each n as stated above.

### 2.4 Deep Learning Analysis

After performing initial LDA analysis for classification, we continued to refine and improve the results of our classification algorithm through the use of deep learning techniques. We employed four different pre-trained convolutional neural network (CNN) architectures to evaluate computational efficiency and algorithm prediction performance among them: VGG-16,^57^ ResNet50,^58^ EfficientNet,^59^ and MobileNet.^60^

For each sample, we combined three MPM channels to create pseudo-RGB images compatible with the input to the CNNs. For the first part of the study using only FFPE samples, we combined NADH, lipofuscin, and SHG, and for the second part of the study using both FFPE and fixed frozen samples we substituted FAD for NADH. Unstitched tiles were used instead of whole stitched images as during our ML analysis in order to increase the number of images provided to the CNN, resulting in 169 tiles for each sample. Each tile was cropped to remove the overlap regions. To further increase dataset size, augmentation techniques such as rotations and reflections were used. 70% of samples were allocated for training, 15% for validation, and 15% for testing. All tiles from each sample were allocated for the same purpose to prevent information leakage.

**Fig. 4:**
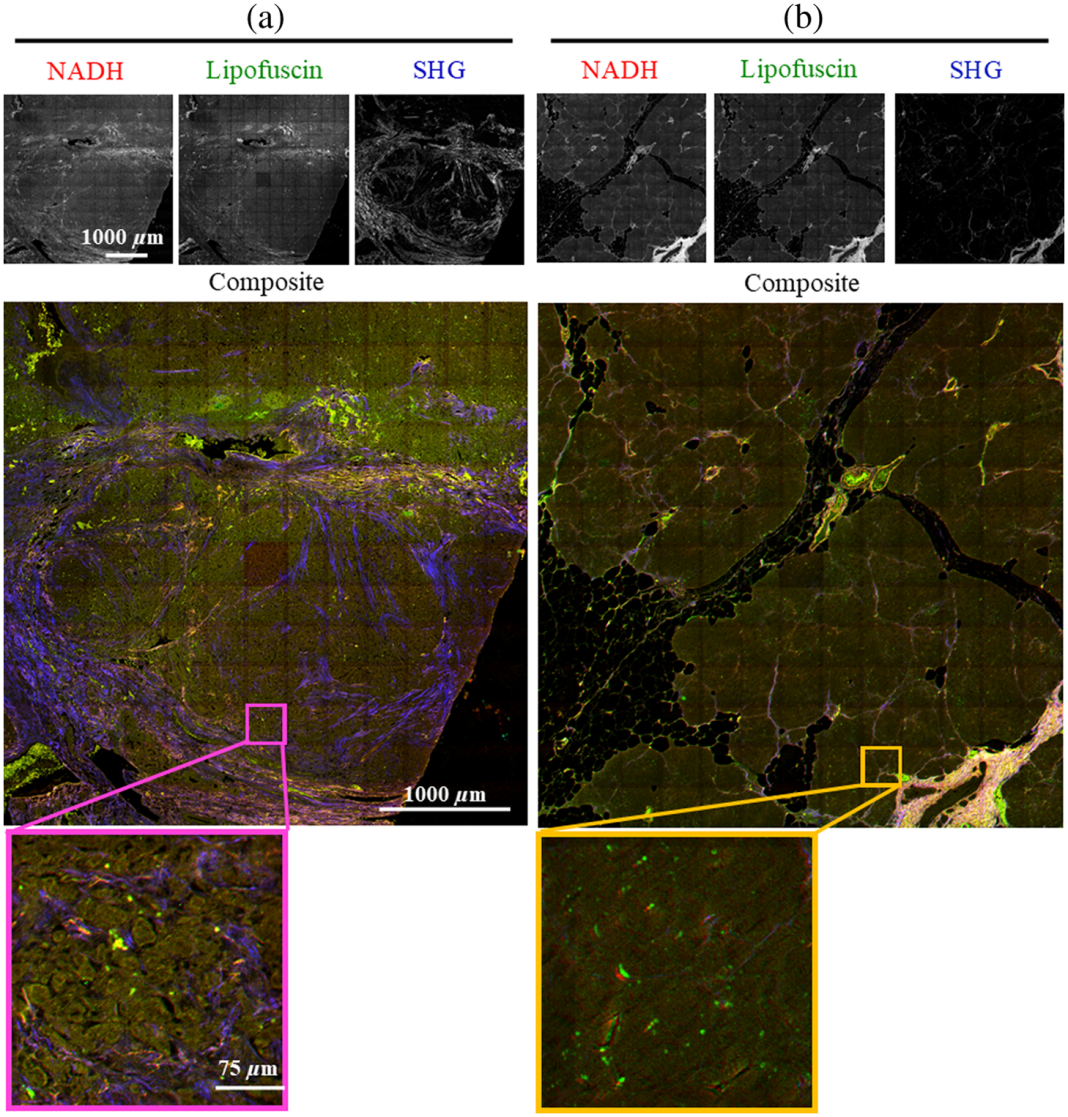
A representative (a) tumor and (b) normal pancreatic tissue FFPE sample. Note that the individual tiles (shown as an ROI on the composite image), which were stitched to make this image, were the actual inputs to the CNN. (R,G,B) = (NADH, Lipofuscin, SHG).

Figure 4 shows a representative whole RGB image, reflecting NADH, lipofuscin, and SHG channels, respectively, for a tumor and normal FFPE sample. An individual tile from each is shown in detail. Note that individual tiles, not the whole image, were the inputs to the CNN, so the apparent tiling artifact was not observed in the input tiles.

**Fig. 5:**
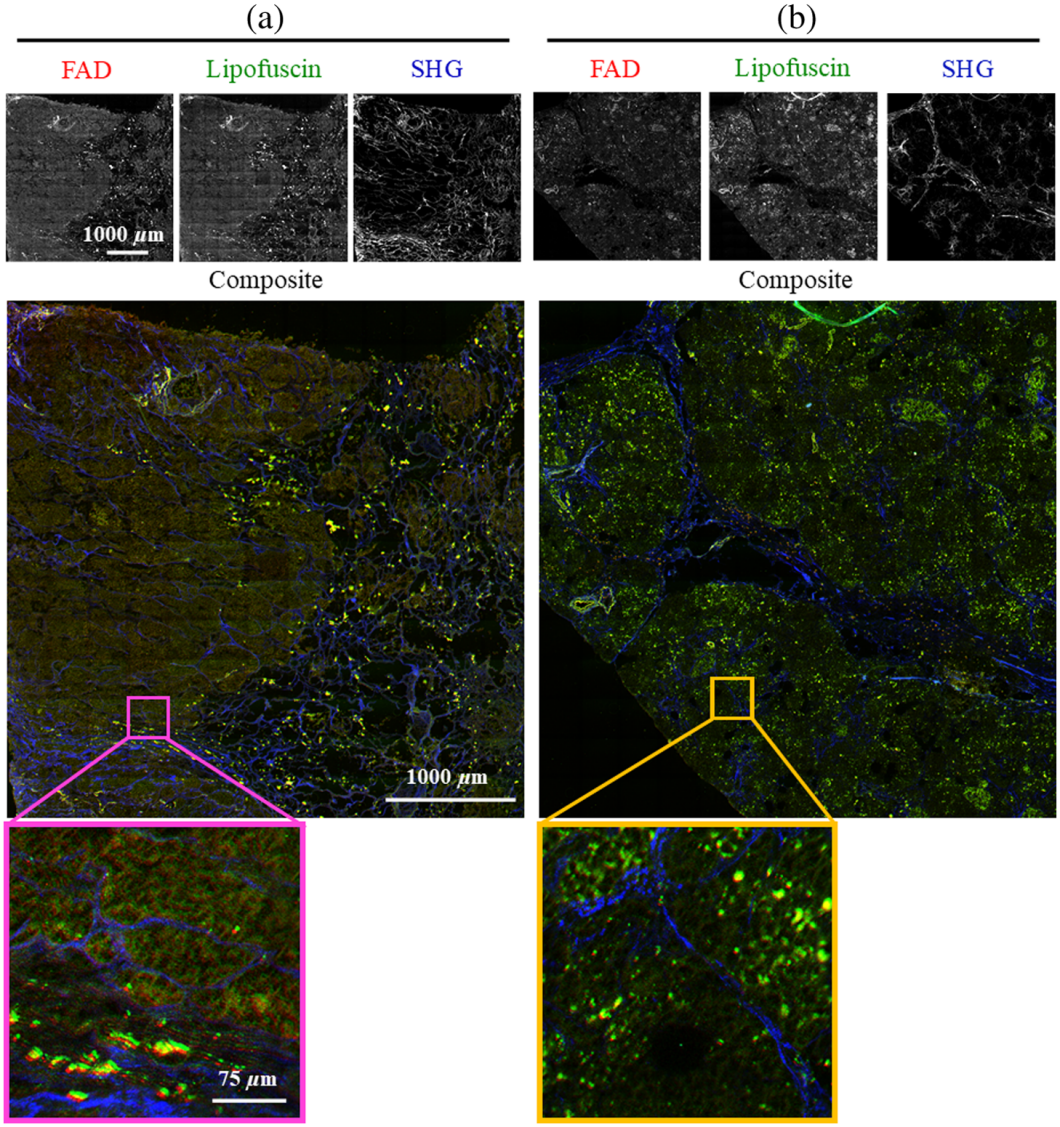
A representative (a) tumor and (b) normal pancreatic tissue fixed frozen sample. Note that the individual tiles (shown as an ROI on the composite image) which were stitched to make this image were the actual inputs to the CNN. (R,G,B) = (FAD, Lipofuscin, SHG).

Figure 5 shows a representative whole RGB image, reflecting FAD, lipofuscin, and SHG channels, respectively, for a tumor and normal fixed frozen sample. The RGB FFPE images shown in Fig. 4 were regenerated with FAD as the red channel to create the dataset for the CNNs for the second part of the study using the combined dataset.

Training of VGG-16, ResNet50, EfficientNet, and MobileNet were carried out on a high-performance computing machine equipped with an NVIDIA GeForce A4500 GPU. Random search was applied here to optimize the hyperparameters for the models. L2 regularization and dropout techniques were applied to the output layer, and early stopping, learning rate scheduler, and 5-fold cross-validation were utilized to address the overfitting and biasing problems. Only the fold with the best performance was used for network training. Furthermore, an assessment of the models’ training times was performed, from which we observed that MobileNet exhibited the fastest training time at less than 20 min, and VGG16 the longest at 2.5 h. Full details of the way these CNNs were fine-tuned to suit the needs of this study, and a more complete review of their training times, can be found in Guan et al.^61^

## 3 Results

### 3.1 Analysis of Handcrafted Features

**Fig. 6:**
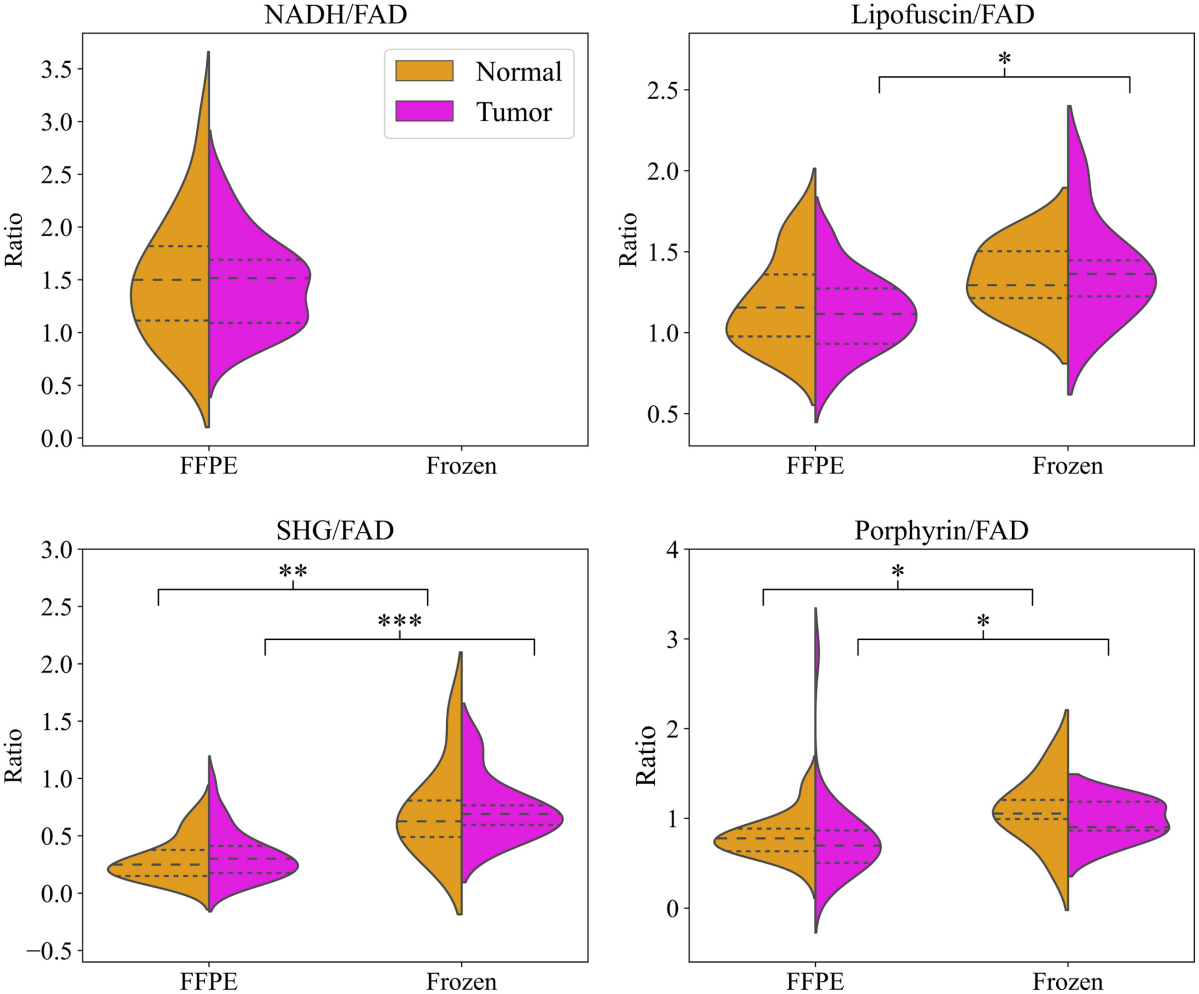
Violin plots showing the distribution of average image brightness across all samples. Note the NADH channel cannot be compared for the fixed frozen dataset due to the interaction with the mounting medium. Any background space in images was masked out and zero values were discarded before averaging to avoid skewing the data. Channels were normalized by FAD brightness. Dashed lines within the plots indicate the mean, and the dotted lines mark the interquartile range. * = p<0.05, ** = p<0.01, *** = p<0.001.

We compared relative image brightness to determine if significant brightness differences occurred between the tumor and normal samples and between the two sample preparations in each of the imaging channels. The average brightness of each sample for each channel was calculated, and then this number was normalized by the average FAD brightness for that sample to account for background. We used the Welch’s t-test or Mann Whitney U test depending on the normality of the distributions, with significance levels at p<0.05, p<0.01, and p<0.001. Note these significance levels are tighter than those we used to analyze the Haralick features below as looser significance levels obscured differences between the populations. The distribution of values can be seen in Fig. 6. While there was no statistically significant difference in brightness between the tumor and normal distributions for any preparation, there was significant difference in brightness between FFPE and fixed frozen tumor samples all channels able to be compared, and significant difference between normal distributions for SHG and porphyrin. In particular, the FFPE and fixed frozen samples differed the most in the SHG channel. We can conclude that fixed frozen samples appear brighter than their counterparts on average, but that there is not significant brightness difference between tumor and normal samples.

**Fig. 7:**
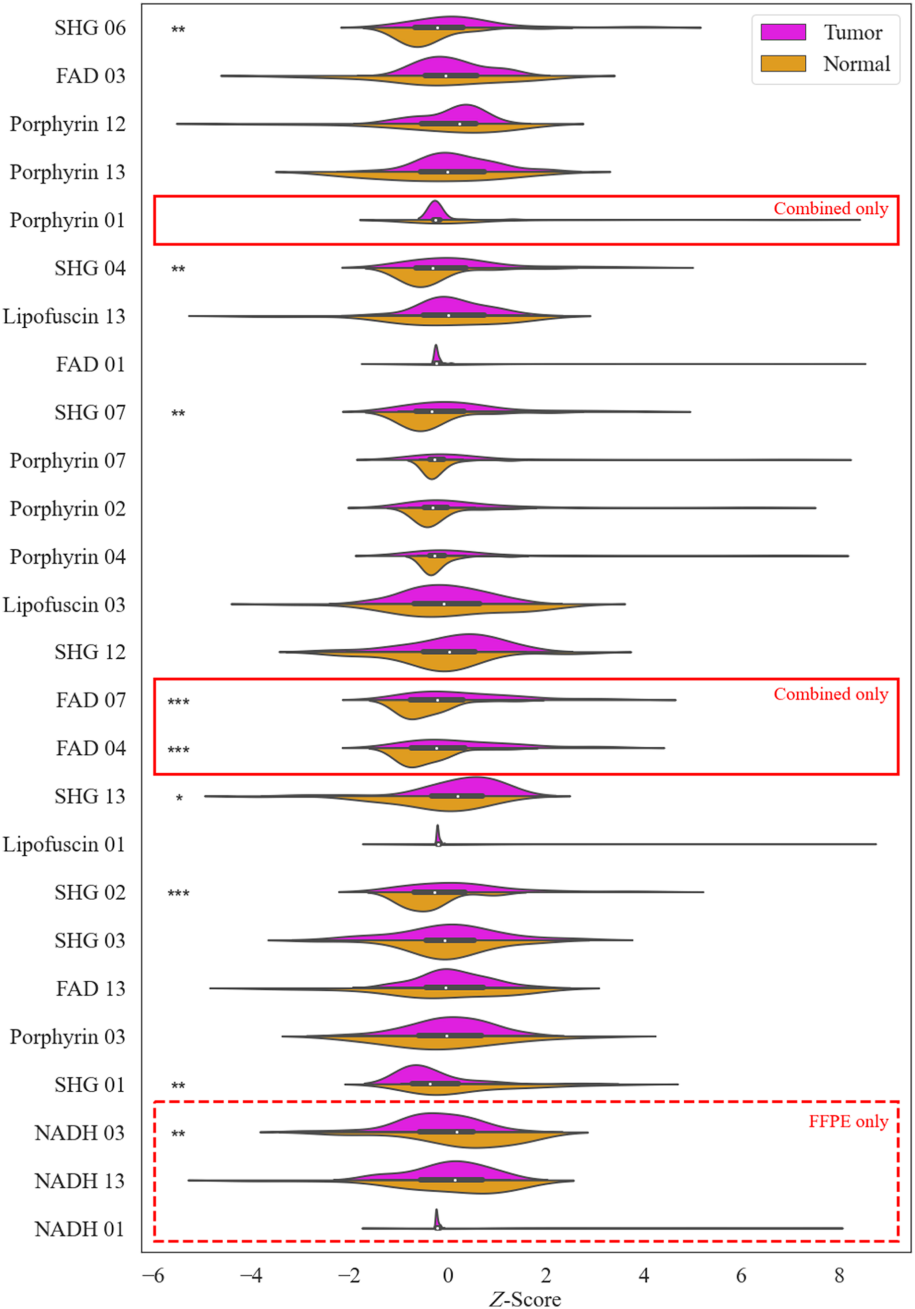
Distributions of the z-scores of the top 23 features for the FFPE and combined datasets. Statistical significance is denoted by the * symbol to the left of the distribution plot. * = p<0.1, ** = p<0.05, *** = p<0.02. Features are ordered from top to bottom according to how often they were chosen by the LDA algorithm over all runs for the combined dataset. Porphyrin 01, FAD 04, and FAD 07 were only used for the combined dataset, and NADH 03, NADH 13, and NADH 01 were only used for the FFPE dataset.

Figure 7 shows the distribution of feature values for the top 23 features which met the correlation threshold in the combined dataset and the FFPE dataset, ordered by the frequency with which they were chosen by LDA for the combined dataset analysis. The top 23 features with low correlation between the FFPE only and the combined dataset are identical with the exception of Porphyrin 1, FAD 4, and FAD 7 in the combined dataset, which are alternatives to NADH 1, NADH 3, and NADH 13 in the FFPE dataset as the NADH channel was unable to be used for the combined dataset. Statistical significance between the tumor and normal samples was evaluated. Nine features had statistically significant difference between the tumor and normal distributions at a threshold of p<0.1, seven had significance at p<0.05, and three had significance at p<0.02. For the FFPE dataset, the features with significance were all in the top half of features selected during ML analysis. However, for the combined dataset the features which were marked significant were not necessarily those which were chosen most often by the algorithm.

Of all channels, the SHG channel was represented the most with eight features in the top 23 features below the correlation threshold. Porphyrin was the next highest at seven, then FAD with five features. Haralick features 1, 3, and 13 were chosen for each of the four channels, features 4 and 7 chosen for three channels, and features 2 and 12 each chosen two times. Features 5, 8, 9,10, and 11 were never chosen. Given that the channels and features represented here are extremely similar to those chosen during the FFPE dataset feature reduction, we can conclude that the addition of the fixed frozen samples did not greatly affect the correlation between features for the combined dataset.

### 3.2 LDA Classification

**Fig. 8:**
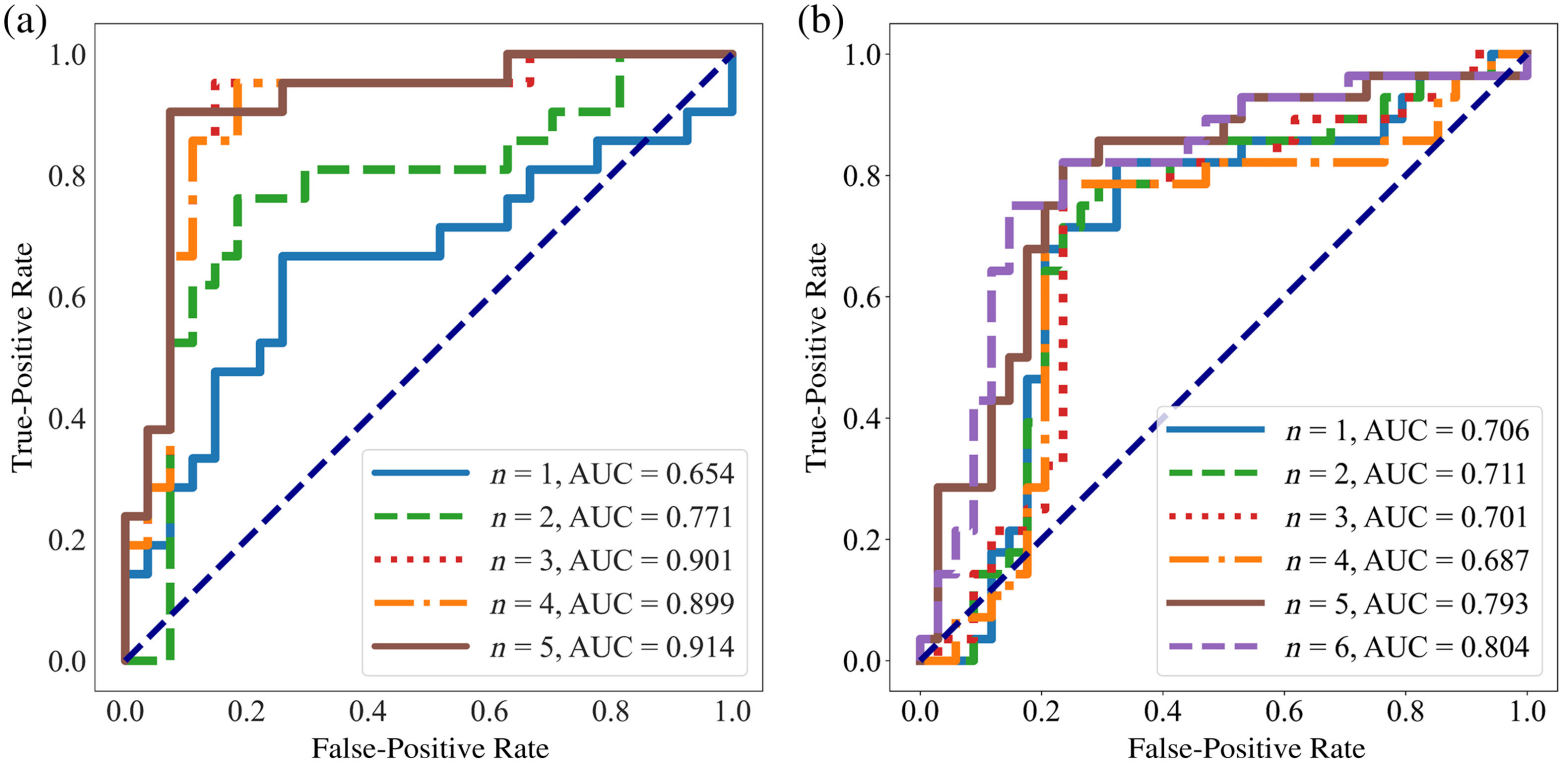
(a) Receiver operator characteristic (ROC) curve showing the results of classifying the FFPE samples using LDA and n=1,2,3,4,5 features. (b) Receiver operator characteristic (ROC) curve showing the results of classifying the combined samples using LDA and n=1,2,3,4,5,6 features. For both plots, the features used for each subset were the top features for that n.

Figure 8(a) shows the results of our ML classification algorithm for various subsets of the above features for the FFPE dataset. As we increased the number of features given to the algorithm to n=5, we achieved a peak AUC value of 0.914. AUC hovers around for 0.9 for n=3,4 features as well. After n=5, algorithm performance plateaued and began to degrade, which suggests overfitting with n>5. Figure 8(b) shows the results of our ML classification algorithm for the combined dataset. As we increased the number of features given to the algorithm to n=5 AUC jumped to 0.793, and at n=6 we achieved a peak value of 0.804. AUC hovers around for 0.7 all other subsets of features. After n=6, algorithm performance plateaued and began to degrade as with the FFPE dataset.

Table 3 shows the mean and standard deviation of the accuracy of the algorithm for each n over 100 runs. For the FFPE dataset, accuracy increases from n=1 to 5 features, and the standard deviation drops to 0.0% at n=4,5 features; this indicates the model behaves more stably as the number of features increases. A peak accuracy of 87.5±0.0% occurs at n=4,5 features. For the combined dataset, accuracy increases from n=1 to 6 features, and the standard deviation drops to 0.0% at n=5 features, but is still below 1% for n=2,3,4. A peak accuracy of 80.6±0.0% occurs at n=6 features.

**Table 3:** Mean accuracy of the LDA algorithm at classifying the combined dataset for each set of best features, averaged over 100 runs.

Number of features	Mean accuracy FFPE dataset	Mean accuracy combined dataset
1	70.5±3.4%	71.9±3.60%
2	76.0±1.5%	74.1±0.45%
3	84.6±1.1%	75.5±0.61%
4	87.5±0.0%	77.1±0.61%
5	87.5±0.0%	77.4±0.00%
6	—	80.6±0.00%

**Fig. 9:**
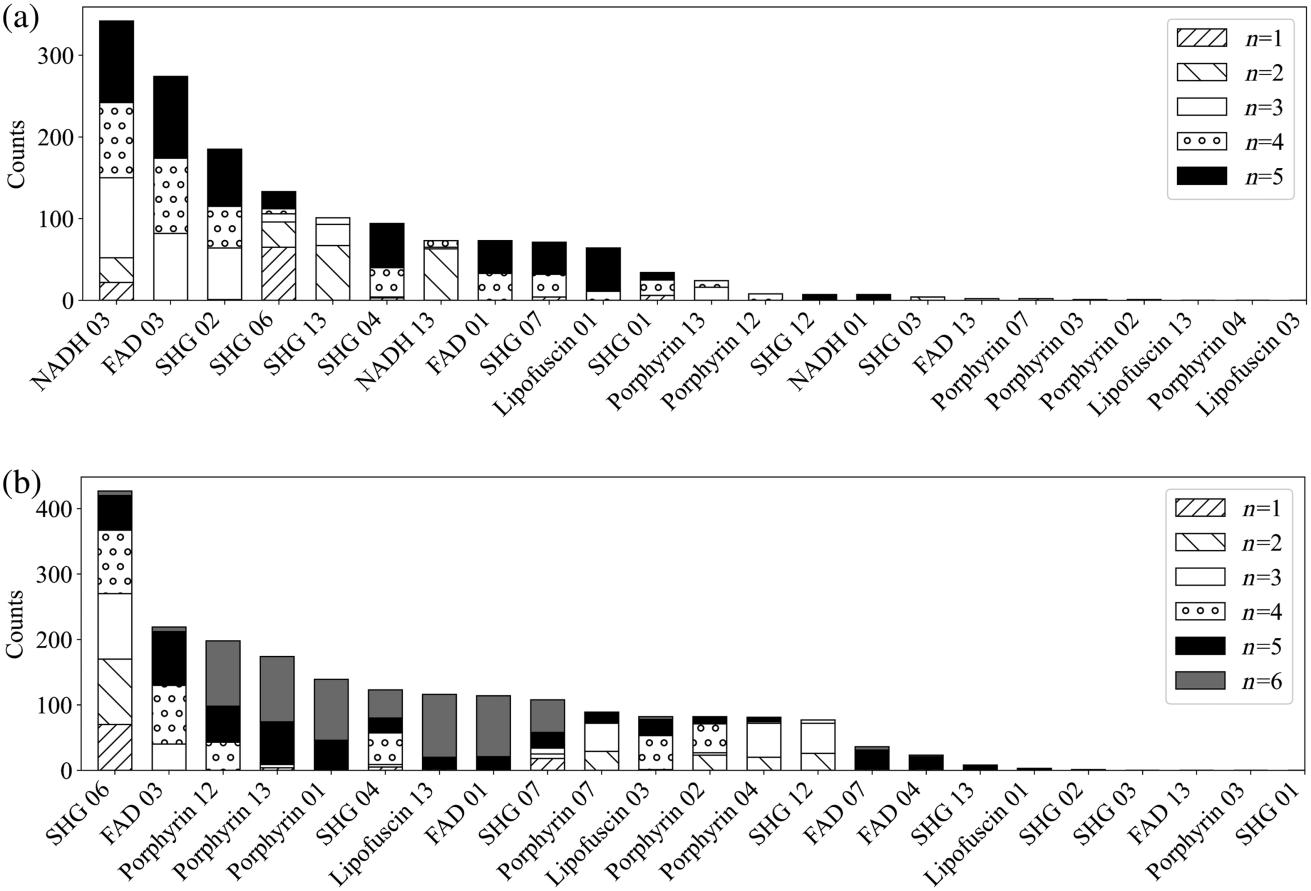
(a) Of the 23 reduced features given to the algorithm to classify the samples, this chart shows which feature was chosen most often by each subset of n features for the FFPE dataset. (b) The feature most chosen by each subset of n features for the combined dataset. LDA was performed for each subset 100 times, so the maximum number of times a feature could theoretically be chosen is 500 for the FFPE dataset, and 600 for the combined dataset. Features are organized from most to least times chosen over all subsets and runs.

Figure 9(a) shows a histogram of feature selection for the FFPE dataset for n=1,2,3,4,5 features. Around half of the 23 total features were chosen with some appreciable frequency, and half were never or rarely chosen. The subsets n=1,2,3 seemed to each strongly favor a particular subset of features. For n=1 this was SHG 06. For n=2 this was SHG 13 and NADH 13, and for n=3 this was NADH 03, FAD 03, and SHG 02. The subsets of n=4,5 features had very similar trends; the three features selected most often for both subsets were NADH 03, FAD 03, and SHG 02, and the remaining selected features were fairly evenly distributed among SHG 04, FAD 01, and SHG 07. Porphyrin and lipofuscin features are rarely chosen; this information validates the selection of channels for the pseudo-RGB images input to the CNNs during deep learning analysis.

Figure 9(b) shows a histogram of feature selection for the combined dataset for n=1,2,3,4,5,6 features. The top feature was frequently chosen, and around half of the 23 total features were chosen with less frequency. Only a few features were never chosen. SHG 06 was chosen nearly every time by n=1,2,3,4, but n=5 instead slightly favored FAD 03. Compared to the features chosen to classify the FFPE only dataset, for each subset there was a larger distribution of features chosen. While n=3,4,5 all chose SHG 06 and FAD 03, the remaining features they chose were not commonly shared.

**Fig. 10:**
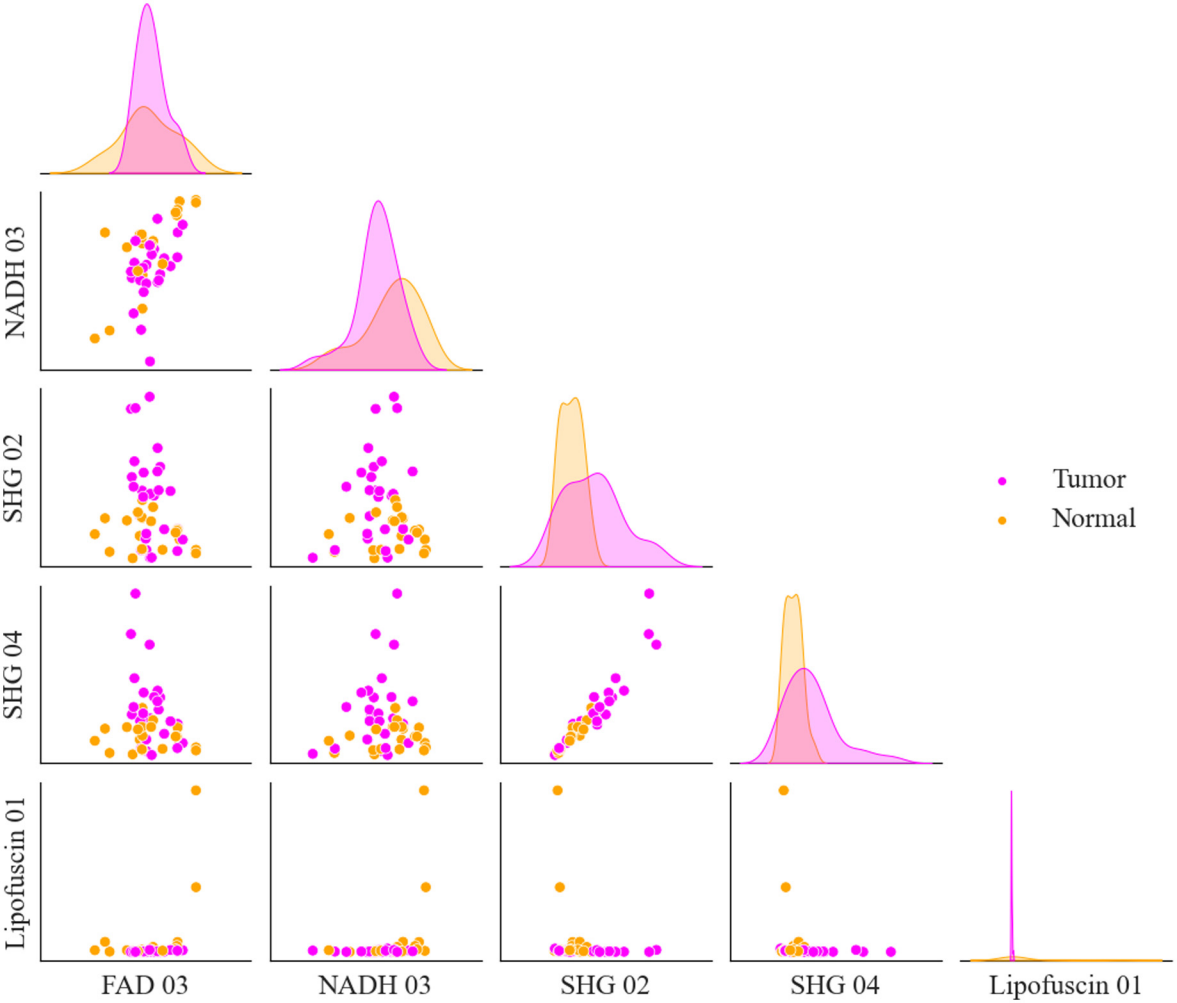
A joint plot showing the feature values for all 48 FFPE samples for top five features selected by the algorithm for the n=5 subset. Tumor samples are shown in magenta, and normal samples in orange. On the off-diagonals, the feature values are plotted against each other, and on the diagonal a histogram of the feature distribution is plotted for the tumor and normal samples separately.

Figure 10 plots the top five features chosen by the algorithm during the FFPE dataset classification for the n=5 subset against each other. This plot provides a visual representation of the LDA process, as the algorithm will create a linear combination of these features and set a threshold to classify the samples based on the information in this plot. Clear clustering of the tumor and normal samples can be seen in the off-diagonal components, particular in the plot of FAD 03 versus SHG 02, where the tumor samples appear clustered in the horizontal direction and the normal samples appear clustered in the vertical direction.

**Fig. 11:**
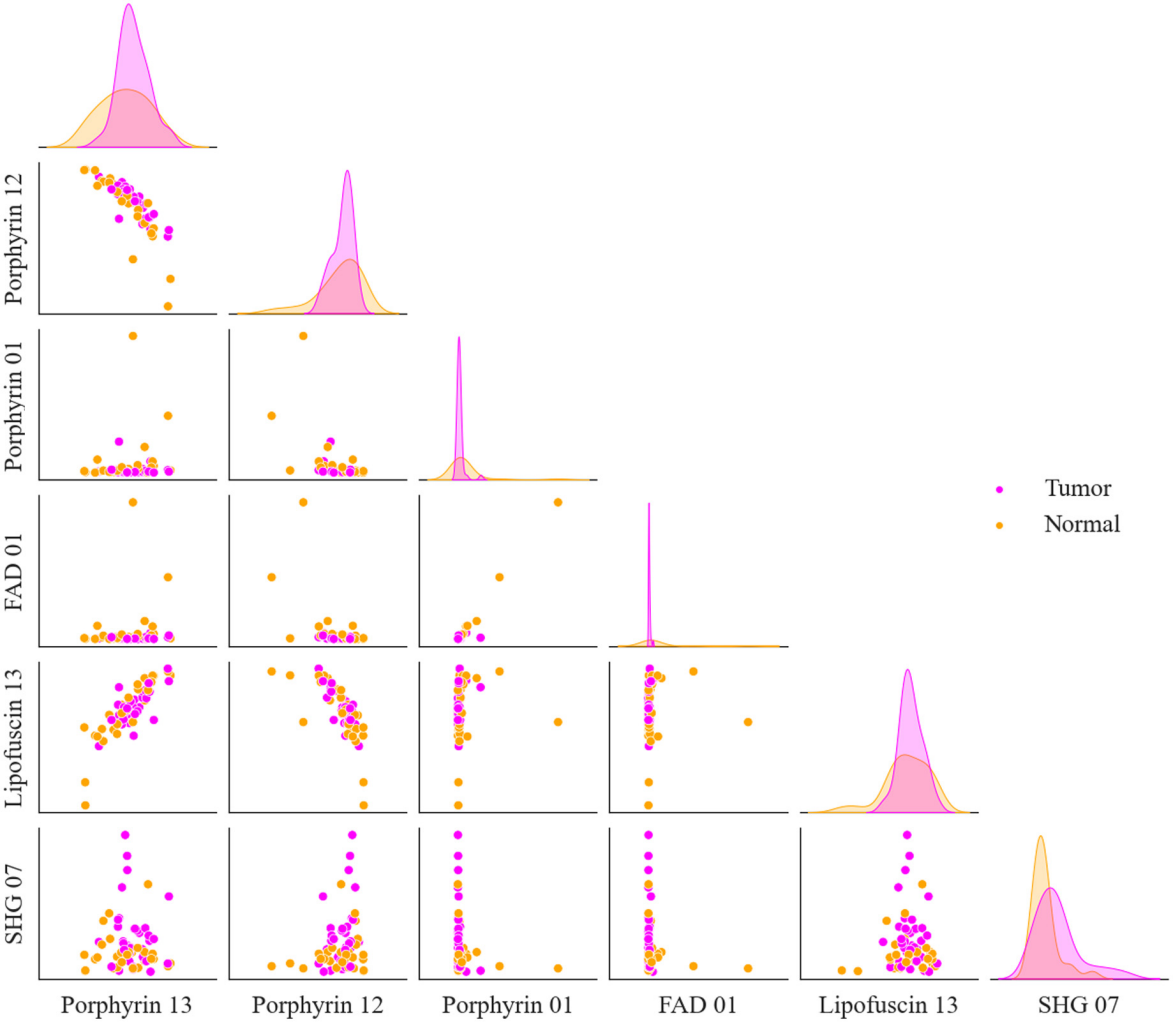
A joint plot showing the feature values for all 62 samples in the combined dataset for top five features selected by the algorithm for the n=5 subset. Tumor samples are shown in magenta, and normal samples in orange. On the off-diagonals, the feature values are plotted against each other, and on the diagonal a histogram of the feature distribution is plotted for the tumor and normal samples separately.

Figure 11 plots the top five features chosen by the algorithm during the combined dataset classification for the n=6 subset against each other. Some clustering of the tumor and normal samples can be seen in the off-diagonal components, although less clearly than for just the FFPE dataset. The most clear clustering can be seen in the SHG 07 versus Porphyrin 13 plot.

**Fig. 12:**
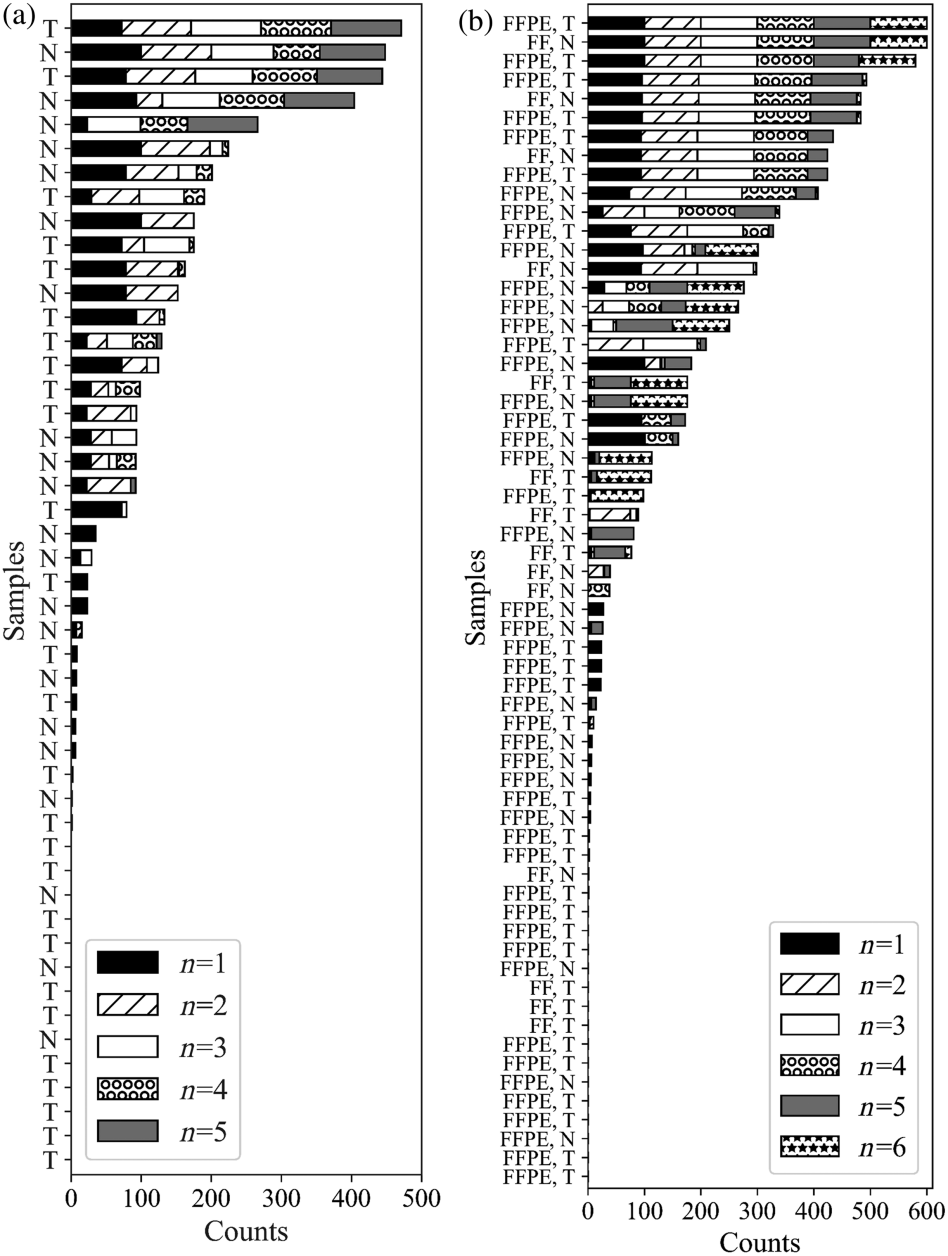
(a) The frequency upon which the 48 FFPE samples were misclassified by the algorithm for n=1,2,3,4,5 features. Tumor samples are marked with a T, and normal samples are marked with an N. (b) How often each of the 62 combined samples were misclassified by the algorithm for n=1,2,3,4,5,6 features. Samples are labeled FFPE for FFPE preparation, FF for fixed frozen preparation, T for tumor, and N for normal samples. LDA was performed for each subset 100 times, so the maximum number of times a sample could theoretically be misclassified is 500 for the FFPE dataset and 600 for the combined dataset. Samples are organized from most to least number of times misclassified over all subsets and runs.

Figure 12(a) shows a histogram of how often each sample was misclassified by the LDA algorithm for each subset of n features across all of its 100 runs for the FFPE dataset. As n increases from 1 to 5, the spread of samples which are misclassified narrows, and the overall number of misclassified samples drops. Four samples are nearly always misclassified, whereas the remaining 44 samples are misclassified half as often or less as the top four. These four samples come from four different patients are not paired tumor and normal sections, although the tumor sections come from the same biorepository. The remaining tumor samples from this repository were not misclassified more frequently than tumor samples from the other two repositories, so this is likely not due to site-specific sample preparation. The tumor samples could potentially be a different subtype of PNEN than the other 25 tumors and have distinct features which make them difficult for the algorithm to classify. Whether a sample is tumor or normal does not seem to have an impact on whether or not it is correctly classified.

Figure 12(b) shows a histogram of how often each sample was misclassified for the combined dataset. Because the accuracy of the algorithm did not significantly increase from n=1 to 6 the number of samples, which are misclassified only decreases slightly as n increases. The top three samples are nearly always misidentified, even by n=6 features. Of these three samples, two are FFPE, but these FFPE samples are not those which were most frequently misclassified during the FFPE only analysis. However, of the top ten misclassified FFPE samples in the combined dataset, six are also present in the top 10 misclassified samples for the FFPE only analysis. Interestingly, from n=1 to n=5 the samples which are incorrectly identified tend to be the same, but at n=6 some samples which were previously nearly always correctly classified are now misclassified. Of the samples which were misclassified at least 100 times, the FFPE:FF ratio is 4.25, which is close to the total sample ratio of 3.5, indicating that sample preparation is not a factor in a sample being correctly identified. Similarly, the ratio of tumor to normal samples misclassified is nearly equal.

### 3.3 Deep Learning

**Fig. 13:**
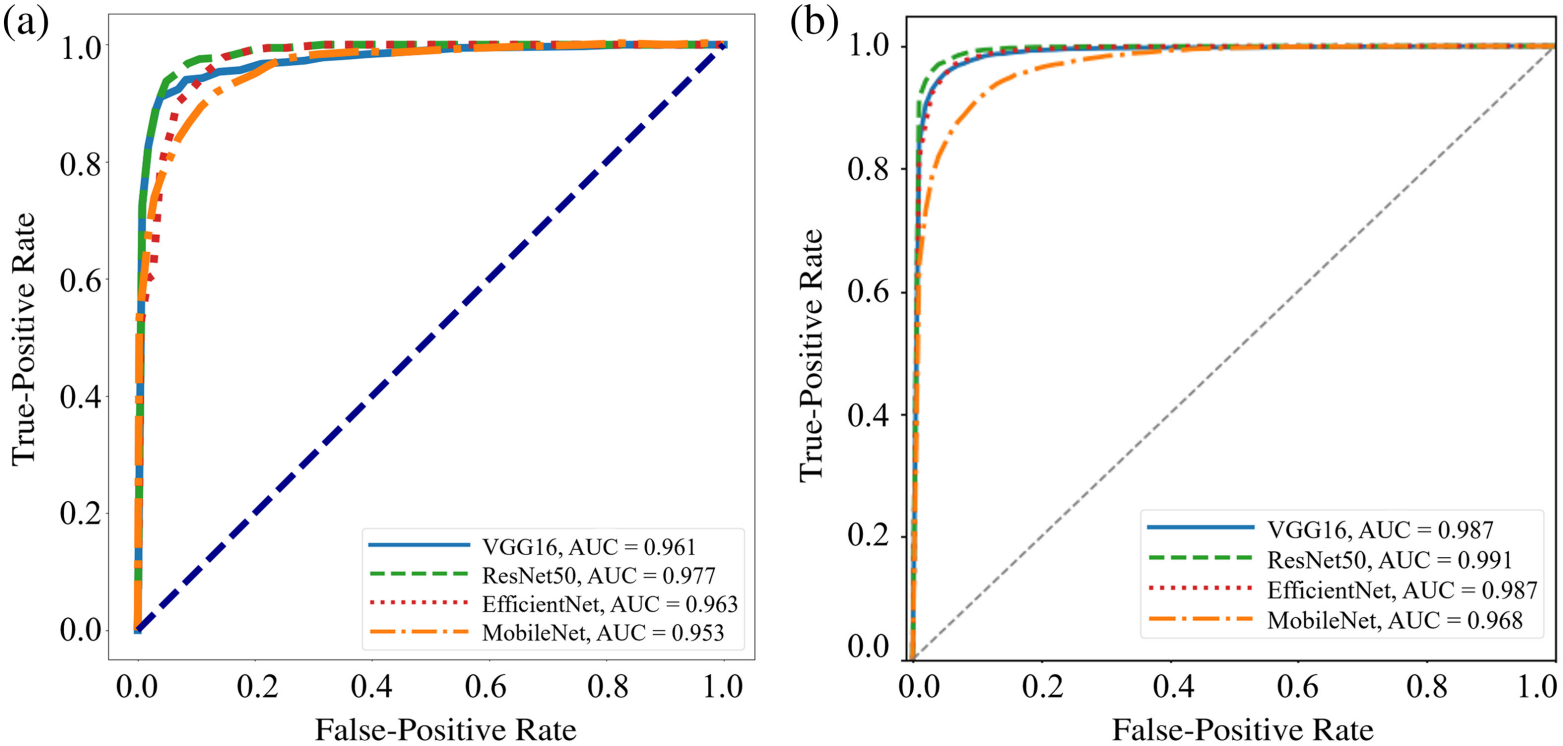
(a) Receiver operator characteristic (ROC) curves of the four CNN models for the FFPE dataset, all with an AUC above 0.950. (b) Receiver operator characteristic (ROC) curves of the four CNN models for the combined dataset, all with an AUC above 0.960. The high AUC values for the testing phase indicate the strong generalization abilities of our models.

Figure 13(a) shows the results of our deep learning classification algorithm for the four CNNs we examined for the FFPE dataset. ResNet50 has the highest mean AUC value at 0.977. VGG16 and EfficientNet have similar values, while MobileNet performs slightly worse. AUC values are always higher than 0.950. Figure 13(b) shows the results for the combined dataset. ResNet50 has the highest AUC value again at 0.991. VGG16 and EfficientNet both have an AUC of 0.987, while MobileNet has an AUC of 0.968. AUC values are higher for every CNN for the combined dataset than for the FFPE dataset.

Table 4 shows the accuracy of the various CNNs. For the FFPE dataset, ResNet50 has the highest accuracy at 94.31%, and MobileNet has the lowest at 88.79%. VGG16 and EfficientNet fall in between, at around 92% accuracy. For the combined dataset, ResNet50 has the highest accuracy again at 96.43%, and MobileNet has the lowest at 90.80%. VGG16 and EfficientNet are both around 95% accuracy.

**Table 4:** Accuracy of the CNNs at classifying the two datasets.

Model	FFPE dataset accuracy (%)	Combined dataset accuracy (%)
VGG16	92.03	95.23
ResNet50	94.31	96.43
EfficientNet	91.42	95.35
MobileNet	88.79	90.80

**Fig. 14:**
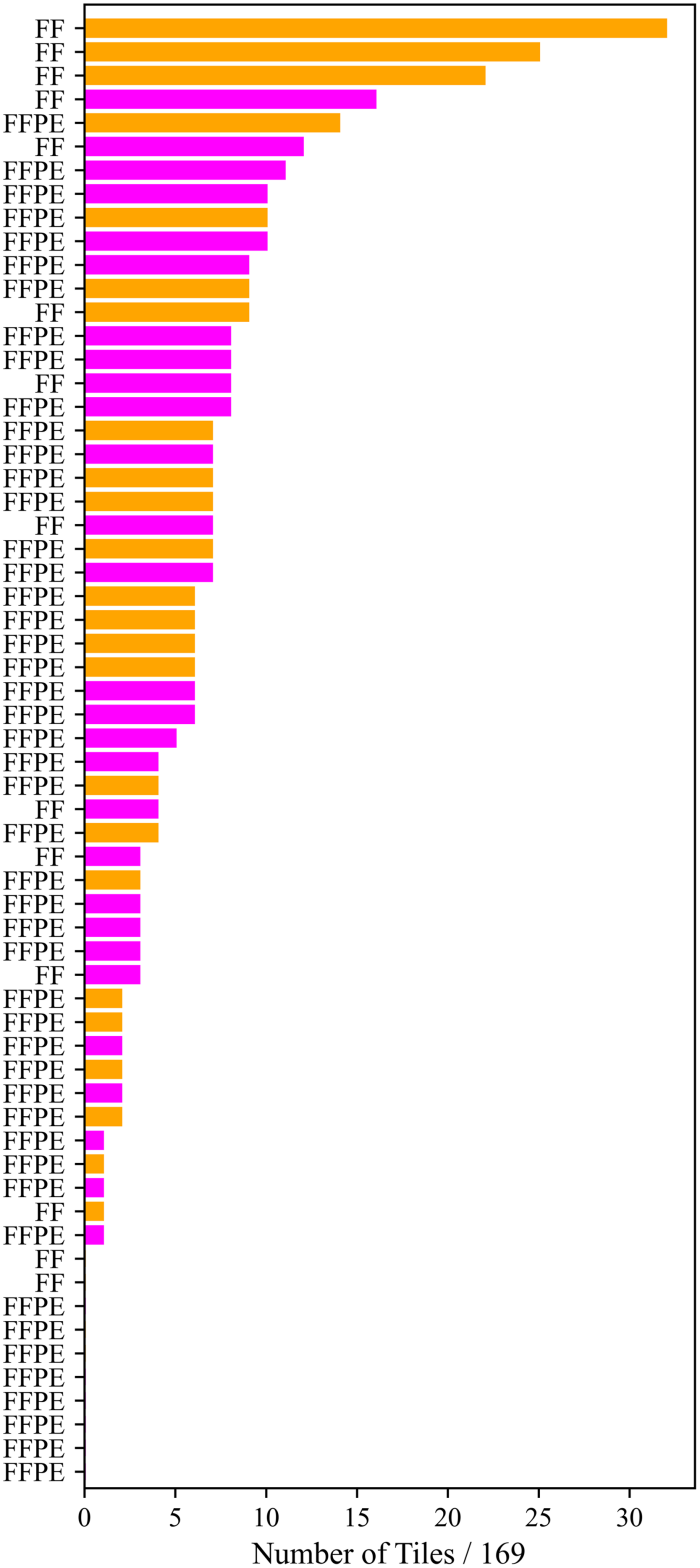
A count of how many tiles of each of the 62 samples from the combined dataset were misclassified by ResNet50. Magenta sample labels indicate tumors, and orange labels indicate normal samples. Each sample had 169 tiles, so the maximum number of times a sample could theoretically be misclassified is 169. Samples are organized from most to least times misclassified over all subsets and runs.

Figure 14 shows a histogram of how many tiles of each sample of the combined dataset was misclassified by ResNet50, the CNN with the highest AUC value and accuracy. Unlike the LDA results, there are trends in which samples are misidentified. Fixed frozen samples are misclassified more often than FFPE samples, and four of the top five most misclassified samples are normal samples. Overall, 6.00% of all fixed frozen tiles and only 2.71% of FFPE tiles were not correctly identified. 2.92% of all fixed tumor tiles and 4.10% of normal tiles were not correctly identified. However, the sample which is incorrectly identified the most only had 32 misclassified tiles out of a possible 169, which constitutes 18.9% of the image. While ResNet50 does seem to favor FFPE and tumor samples, even samples which have misclassified tiles are over 80% correctly classified across the whole image.

For the combined dataset, we performed backprojection on the results of the ResNet50 CNN to determine which samples were misclassified. Supplementary Table 2 shows these results compared to samples which were misclassified by the LDA algorithm with n=6 features for the combined dataset. There is no correlation between which samples were incorrectly identified by ResNet50 and the LDA algorithm; samples which were always misclassified by LDA had at most 10 tiles misclassified by ResNet50, and the sample misclassified by ResNet50 most often was only misclassified by LDA seven times out of 100 runs.

## 4 Discussion

In the first part of the study, we were able to effectively classify the FFPE and combined datasets using ML. For the FFPE dataset, we achieved a peak classification accuracy of 89.6% and a peak AUC value of 0.914. For the combined dataset, the LDA algorithm achieved a peak AUC value of 0.804 and a peak accuracy of 80.6%. Then using deep learning, we reported higher peak accuracies and AUC values for the two datasets across four different CNNs. For the FFPE dataset, AUC values were greater than 0.950 in all cases, and classification accuracy peaked at 94.31%. Of the four CNNs, ResNet50 performed the best in terms of AUC value and accuracy. For the combined dataset, ResNet50 again performed the best of any CNN with an AUC value of 0.977 and an accuracy of 96.43%.

The addition of the fixed frozen samples somewhat degraded the performance of the LDA algorithm, but the deep learning algorithm was able to perform with better accuracy compared to the FFPE only dataset. This could be because the increased variation in the dataset due to the fixed frozen samples introduced features in the tumor and normal populations that the algorithm did not extract with the FFPE only dataset; CNNs are able to extract and utilize a greater range of image features than can realistically be considered with traditional ML approaches. Although ResNet50 misclassified fixed frozen and normal tiles more often than FFPE or tumor tiles, no one sample had more than 19% of its total tile set incorrectly identified. The high performance of our algorithms, particularly using a set of samples from various biorepositories, indicates that MPM has high sensitivity as an imaging modality for PNENs compared to normal pancreatic tissue. Furthermore, we confirmed that two common sample preparation types, FFPE and fixed frozen, did not strongly affect the ability of our deep learning algorithms to correctly classify PNENs.

Taking a closer look at trends in LDA results, we note that Haralick features which both had low correlation and were selected with the highest frequency by the LDA algorithm were most often in the SHG channel, and were typically Haralick features 2, 3, 4, 6, 12, and 13. Features 2 and 3 are contrast and correlation, and are related to the frequency and magnitude of pixel value changes across the image; similarly, feature 4 is the sum of squares: variance, and is indicative of contrast across the whole image rather than on a pixel to pixel basis as with feature 2. Feature 6 is the sum average, and is tied to overall image brightness. Features 12 and 13 are paired features known as the information measures of correlation, and are intended to probe statistical correlation and dependence of information contained in the image GLCMs.^62^ We can conclude that the SHG channel and the ideas of contrast and correlation within image data are important factors during LDA classification. Compared to the CNNs, LDA gives the user more control over the information put into the algorithm and is more transparent about how decisions are made. This provided valuable insight into quantitative differences between MPM images of PNENs and normal tissue, but ultimately deep learning produces more accurate results. All four of the CNNs tested performed better than LDA for any number of features, suggesting that the hierarchical feature extraction inherent to CNNs is more powerful than features extracted through single scale Haralick feature extraction. In future work, comparing relative performance across other feature extraction methods and classification approaches would give more insight into the factors which influence algorithm performance.

Overall, results from this study indicate that MPM as an imaging modality can provide high sensitivity in distinguishing PNENs from normal tissue in histopathological sections. Brightness alone is not enough to definitively classify tissue, but the Haralick spatial texture features contain more information than brightness, and in combination with ML techniques, can classify tissue with increased sensitivity. CNNs offer an even higher level of sensitivity likely due to their hierarchical feature extraction. Imaging time was roughly 2 h for each sample in this study, but with further optimization of imaging parameters, wavelength selection, and field of view, this time could be further reduced. While our approach is faster than standard histological processing, further optimization would allow for significantly reduced processing time, making a more significant impact on the histopathological workflow. In future work, we plan to image fresh, unprepared tissues to evaluate the efficacy of MPM in imaging pancreatic tissue in a surgical setting and establish the reproducibility of our results. Although we did not observe any dependence on our LDA algorithm to sample preparation, these results would be strengthened by confirming our ability to classify unprocessed tissue. Over time, the acquisition of more PNEN samples will also allow us to probe whether the grade and type of PNEN can be ascertained from MPM images using deep learning, something that is not currently possible with our limited dataset.

## 5 Conclusion

In this study, we performed multiphoton imaging of PNENs and normal pancreatic tissue samples, and employed ML and deep learning computational analysis techniques to examine inherent differences in these images. Using LDA we developed classifiers which can classify PNENs from normal tissue with over 80% accuracy, and using CNNs we are able to develop classifiers with over 96% accuracy. Furthermore, fast training times and the subsequent quick decision-making ability of trained CNNs show promise for the future development of real-time classification of PNENs using MPM images. We demonstrate that our algorithms perform well within and across two distinct preparations, providing strong evidence that MPM in combination with deep learning may be a path toward automated pathology to provide faster diagnosis and margin analysis of these tissues.

## Supplementary Material

10.1117/1.BIOS.2.4.045001.s01

## Data Availability

Data is being prepared for access and will be posted as soon as it is available.
