# Supplementary material for: Investigating machine learning algorithms to classify label-free images of pancreatic neuroendocrine neoplasms
Source: Biophotonics Discov. 2025 Oct 2;2(4):045001. doi: 10.1117/1.BIOS.2.4.045001 (PMC13101064; doi:10.1117/1.BIOS.2.4.045001)
Supplement: Supplementary file 1 [file BIOS_002_045001_SD001.pdf]

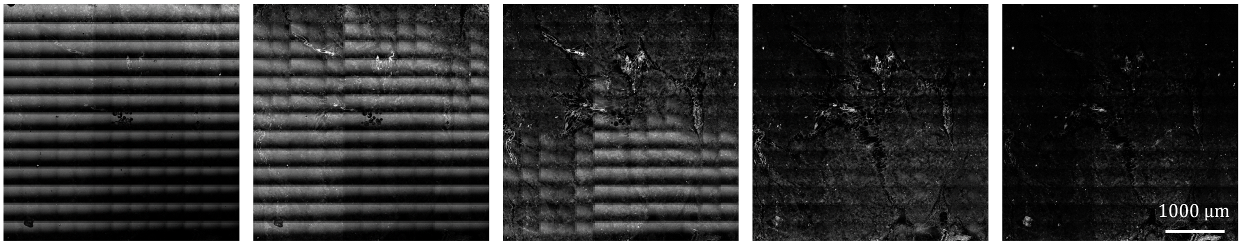


**Supplementary Figure 1** Multiphoton images of the NADH channel for one sample at five depths. $Z = 0\mu m$ is the focal plane centered on the tissue. As the imaging depth moves above the tissue ($Z = -4\mu m$, $Z = -2\mu m$), we see a glare artifact due to fluorescence from the mounting medium atop the sample. As the imaging depth moves below the tissue ($Z = +2\mu m$, $Z = +4\mu m$), the glare artifact disappears as expected. However, the sample signal also decreases as we are no longer at the correct depth to excite the tissue.

| Feature | Equation | Feature | Equation |
|---|---|---|---|
| Angular Second Moment | $f_1 = \sum_i \sum_j \{p(i,j)\}^2$ | Sum Entropy | $f_8 = -\sum_{i=2}^{2N_g} p_{x+y}(i)\log(p(i,j))$ |
| Contrast | $f_2 = \sum_{n=0}^{N_{g-1}} n^2 \sum_{i=1}^{N_g} \sum_{j=1}^{N_g} p(i,j)$, $\lvert i-j \rvert = n$ | Entropy | $f_9 = -\sum_i \sum_j p(i,j)\log(p(i,j))$ |
| Correlation | $f_3 = \frac{\sum_i \sum_j (ij)p(i,j) - \mu_x \mu_y}{\sigma_x \sigma_y}$ | Difference Variance | $f_{10} =$ variance of $p_{x-y}$ |
| Sum of Squares | $f_4 = \sum_i \sum_j (i-\mu)^2 p(i,j)$ | Difference Entropy | $f_{11} = -\sum_{i=0}^{N_{g-1}} p_{x-y}(i)\log\{p_{x-y}(i)\}$ |
| Inverse Difference Moment | $f_5 = \sum_i \sum_j \frac{1}{1+(i-j)^2} p(i,j)$ | Information Measures of Correlation | $f_{12} = \frac{HXY - HXY1}{\max\{HX, HY\}}$ |
| Sum Average | $f_6 = \sum_{i=2}^{2N_g} i p_{x+y}(i)$ | Information Measures of Correlation | $f_{13} = (1 - \exp[-2.0(HXY2 - HXY)])^{1/2}$ |
| Sum Variance | $f_7 = \sum_{i=2}^{2N_g} (i - f_8)^2 p_{x+y}(i)$ | Maximal Correlation Coefficient | $f_{14} =$ (Second largest eigenvalue of Q)$^{1/2}$ |

Definitions

$p(i,j) = P(i,j)/R$, the $(i,j)$th entry in a GLCM $P(i,j)$ after normalizing by its maximum value $R$

$p_x(i) = \sum_{j=1}^{N_g} p(i,j)$, the $i$th entry in the marginal probability matrix found by adding the rows of $p(i,j)$

$N_g$ = number of distinct gray levels in the quantized image; for example, $N_g = 255$ for an 8-bit image

$p_y(j) = \sum_{i=1}^{N_g} p(i,j)$

$\sum_i = \sum_i^{N_g}$, and similarly $\sum_j = \sum_j^{N_g}$

$p_{x+y}(k) = \sum_{\substack{i=1 \\ i+j=k}}^{N_g} \sum_{j=1}^{N_g} p(i,j)$, where $k = 2, 3, \cdots, 2N_g$

$p_{x-y}(k) = \sum_{\substack{i=1 \\ |i-j|=k}}^{N_g} \sum_{j=1}^{N_g} p(i,j)$, where $k = 0, 1, \cdots, N_g - 1$

$HXY = -\sum_i \sum_j p(i,j)\log(p(i,j))$

$HX$ = entropy of $p_x$, and similarly $HY$ = entropy of $p_y$

$HXY1 = -\sum_i \sum_j p(i,j)\log(p_x(i)p_y(j))$

$HXY2 = -\sum_i \sum_j p_x(i)p_y(j)\log(p_x(i)p_y(j))$

$Q(i,j) = \sum_k \frac{p(i,k)p(j,k)}{p_x(i)p_y(k)}$

**Supplementary Table 1** The 14 Haralick texture features.[?]

| Sample | Tissue Type | Class | Number of Tiles Misclassified by ResNet50 | Number of Times Misclassified by LDA |
|---|---|---|---|---|
| 1 | Frozen | normal | 32 | 7 |
| 2 | Frozen | normal | 25 | 0 |
| 3 | Frozen | normal | 22 | 0 |
| 4 | Frozen | tumor | 16 | 11 |
| 5 | FFPE | normal | 14 | 0 |
| 6 | Frozen | tumor | 12 | 0 |
| 7 | FFPE | tumor | 11 | 0 |
| 8 | FFPE | tumor | 10 | 0 |
| 9 | FFPE | normal | 10 | 0 |
| 10 | FFPE | tumor | 10 | 100 |
| 11 | Frozen | normal | 9 | 0 |
| 12 | FFPE | normal | 9 | 0 |
| 13 | FFPE | tumor | 9 | 100 |
| 14 | FFPE | tumor | 8 | 0 |
| 15 | Frozen | tumor | 8 | 100 |
| 16 | FFPE | tumor | 8 | 0 |
| 17 | FFPE | tumor | 8 | 0 |
| 18 | FFPE | tumor | 7 | 0 |
| 19 | FFPE | normal | 7 | 93 |
| 20 | Frozen | tumor | 7 | 0 |
| 21 | FFPE | normal | 7 | 100 |
| 22 | FFPE | normal | 7 | 100 |
| 23 | FFPE | tumor | 7 | 0 |
| 24 | FFPE | normal | 7 | 93 |
| 25 | FFPE | tumor | 6 | 0 |
| 26 | FFPE | tumor | 6 | 0 |
| 27 | FFPE | normal | 6 | 0 |
| 28 | FFPE | normal | 6 | 7 |
| 29 | FFPE | normal | 6 | 0 |
| 30 | FFPE | normal | 6 | 0 |
| 31 | FFPE | tumor | 5 | 0 |
| 32 | FFPE | normal | 4 | 0 |
| 33 | Frozen | tumor | 4 | 0 |
| 34 | FFPE | normal | 4 | 0 |
| 35 | FFPE | tumor | 4 | 93 |
| 36 | Frozen | tumor | 3 | 96 |
| 37 | FFPE | tumor | 3 | 0 |

| | | | | |
|---|---|---|---|---|
| 38 | FFPE | tumor | 3 | 0 |
| 39 | FFPE | tumor | 3 | 0 |
| 40 | FFPE | normal | 3 | 93 |
| 41 | Frozen | tumor | 3 | 0 |
| 42 | FFPE | normal | 2 | 0 |
| 43 | FFPE | tumor | 2 | 0 |
| 44 | FFPE | normal | 2 | 0 |
| 45 | FFPE | tumor | 2 | 7 |
| 46 | FFPE | normal | 2 | 0 |
| 47 | FFPE | normal | 2 | 100 |
| 48 | FFPE | tumor | 1 | 0 |
| 49 | Frozen | normal | 1 | 0 |
| 50 | FFPE | tumor | 1 | 0 |
| 51 | FFPE | normal | 1 | 0 |
| 52 | FFPE | tumor | 1 | 0 |
| 53 | FFPE | tumor | 0 | 0 |
| 54 | FFPE | tumor | 0 | 7 |
| 55 | FFPE | tumor | 0 | 0 |
| 56 | FFPE | tumor | 0 | 0 |
| 57 | FFPE | tumor | 0 | 0 |
| 58 | FFPE | normal | 0 | 4 |
| 59 | FFPE | normal | 0 | 0 |
| 60 | FFPE | tumor | 0 | 0 |
| 61 | Frozen | normal | 0 | 0 |
| 62 | Frozen | normal | 0 | 100 |

**Supplementary Table 2** Comparison of samples of the combined dataset which were misclassified by LDA algorithm with $n = 6$ features versus ResNet50. The total number of times a sample can be misclassified by LDA is 100. The total number of tiles which can be misclassified by ResNet50 is 169.
